# Benzylpenicillin-producing *Trichophyton erinacei* and methicillin resistant Staphylococcus aureus carrying the *mec*C gene on European hedgehogs – A pilot-study

**DOI:** 10.1186/s12866-021-02260-9

**Published:** 2021-07-15

**Authors:** Faruk Dube, Robert Söderlund, Matilda Lampinen Salomonsson, Karin Troell, Stefan Börjesson

**Affiliations:** 1 Department of Biomedical Science and Veterinary Public Health, Swedish University of Agricultural Sciences, 75007 Uppsala, Sweden; 2 Department of Microbiology, National Veterinary Institute, SVA, 75189 Uppsala, Sweden; 3 Department of Chemistry, Environment and Feed Hygiene, National Veterinary Institute, SVA, 75189 Uppsala, Sweden; 4 Department of Medicinal Chemistry, Faculty of Pharmacy, Uppsala University, 75123 Uppsala, Sweden; 5 Department of Medical Biochemistry and Microbiology, Uppsala University, 75123 Uppsala, Sweden; 6 Department of Animal Health and Antimicrobial Strategies, National Veterinary Institute (SVA), 75189 Uppsala, Sweden; 7 Department of Microbiology, Public Health Agency of Sweden, 17182 Solna, Sweden

**Keywords:** Antibiotics, Benzylpenicillin, Dermatophytes, Hedgehogs, MRSA, *Staphylococcus aureus*, *Trichophyton erinacei*, *Europeaus erineaus*, Wildlife

## Abstract

**Background:**

A high carriage rate of methicillin-resistant *Staphylococcus aureus* with the *mec*C gene (*mec*C-MRSA) has been described among Wild European hedgehogs (*Europeaus erineaus*). Due to this frequent occurrence, it has been suggested that hedgehogs could be a natural reservoir for *mec*C-MRSA. However, the reason why hedgehogs carry *mec*C-MRSA remains unknown, but it has been hypothesized that *mec*C-MRSA could have evolved on the skin of hedgehogs due to the co-occurrence with antibiotic producing dermatophytes. The aim of this pilot-study was therefore to investigate if hedgehogs in Sweden carry *Trichophyton spp*. and to provide evidence that these dermatophytes are able to produce penicillin or similar substances. In addition, the study aimed to identify if dermatophytes co-occurred with *mec*C-MRSA.

**Methods:**

Samples were collected from hedgehogs (*Europeaus erineaus*) that were euthanized or died of natural causes. All samples were screened for dermatophytes and *mec*C-MRSA using selective cultivation methods. Suspected isolates were characterized using PCR-based methods, genome sequencing and bioinformatic analyses. Identification of penicillin was performed by ultra-high-performance liquid chromatography-tandem mass spectrometry.

**Results:**

In total 23 hedgehogs were investigated, and it was shown that two carried *Trichophyton erinacei* producing benzyl-penicillin, and that these hedgehogs also carried *mec*C-MRSA. The study also showed that 60% of the hedgehogs carried *mec*C-MRSA.

**Conclusion:**

The pilot-study demonstrated that *Trichophyton erinacei,* isolated from Swedish hedgehogs, can produce benzylpenicillin and that these benzylpenicillin-producing *T. erinacei* co-occurred with *mec*C-MRSA. The study also reconfirmed the high occurrence of *mec*C-MRSA among hedgehogs.

## Background

*Staphylococcus aureus* is a commensal of humans and many animals, but it is also an opportunistic pathogen [1]. However, due to the spread of antibiotic resistant strains the ability to treat *S. aureus* infections has been undermined, and of particular concern is the emergence of methicillin-resistant *S. aureus* (MRSA). MRSA has low sensitivity for all clinically used beta-lactam antibiotics, except the fifth generation cephalosporins [2]. When MRSA first emerged in the 1980s it was primarily associated with hospital infections (HA-MRSA), but soon after reports on community associated infections (CA-MRSA) occurred [3]. Today MRSA also occurs in animals and has spread particularly among farm animals, primarily pigs, where occurrence mainly is due to asymptomatic carriage of some specific livestock-adapted clonal linages (LA-MRSA) [4].

All MRSA were originally associated with the *mec*A gene (*mec*A-MRSA), which encodes for an altered penicillin-binding protein (PBP), PBP2a [5]. However, in 2011, a *mec*A homologue, *mec*A_LGA251,_ was identified with about 69% nucleotide identity to *mec*A that codes for an alternative PBP called PBP2c [6]. The *mec*A_LGA251_ gene was later renamed *mec*C (*mec*C-MRSA) and *mec*C-MRSA was first described from bulk milk samples in UK and human samples in Denmark, UK, and Ireland [6, 7]. Subsequent studies have described *mec*C-MRSA from a diverse set of regions and animal species, but almost exclusively from Europe [8–13]. Overall, there appears to be a low incidence of *mec*C-MRSA in most sectors, including in human healthcare settings, but an increasing trend in human clinical cases has been observed in Denmark [14]. At first domestic animals in Europe, primarily dairy cattle, were linked to *mec*C-MRSA and it was therefore suggested that dairy cattle could be the original reservoir [15, 16]. However, the wide distribution of *mec*C-MRSA in different wild animal species challenged that narrative and it was therefore suggested that rodents, insectivores, or small carnivores could be the origin of *mec*C-MRSA [17]. This hypothesis appears too be supported by a Swedish study showing a high occurrence of *mec*C-MRSA, 64%, in wild European hedgehogs (*Europeaus erineaus*) in 2017 [8]. A recent nationwide study in Denmark further strengthens that *mec*C-MRSA is associated with hedgehogs as it showed a carriage rate of 61% [11]. In Sweden, all MRSA are notifiable to the authorities and only 132 instances of MRSA from animals, not counting hedgehogs, have been reported between 2006 and through 2018. For comparison, 3864 MRSA cases were reported from humans in 2018 alone [18]. Cases with *mec*C-MRSA has remained rare in Sweden both among humans and animals, with only 92 and 14 cases respectively reported since 2011 and up to 2018, when excluding hedgehogs. The first *mec*C-MRSA in Sweden was isolated already in 2003 from a hedgehog suffering from *S. aureus* septicemia but it was not described as such until 2012 [19]. After the first report three *mec*C-MRSA isolates were reported from dairy cows in 2010, and since 2011 it has been reported from cats, dogs, dairy cows, dairy and pet goats, and humans [18, 20].

In the 1960s a study conducted by Smith and Marples [21] reported a high proportion, 86%, of penicillin-resistant *S. aureus* in wild European hedgehogs (*E. erineaus*) in New Zealand. This study also noted that 45% of hedgehogs had chronic mycotic infections caused by dermatophytes. Since an earlier study had demonstrated penicillin-like production by dermatophytes [22], Smith and Marples hypothesized that hedgehogs could be a potential natural reservoir of penicillin-resistant *S. aureus* and that antibiotics produced by their dermatophytes could have selected for this resistance phenotype. Subsequently, they were able to show that *Trichophyton spp.* dermatophytes from hedgehogs’ skin likely produce a penicillin-like substance [23].

Based on the described high occurrence of *mec*C-MRSA and *Trichophyton spp.* on hedgehogs and that the dermatophytes have been suggested to produce penicillin-like substances we aimed to investigate if Swedish hedgehogs carry *Trichophyton spp.* that produce penicillin or a similar substance, and if *Trichophyton* spp. co-occurs with MRSA.

## Results

### Recovery and characterization of dermatophytes

**Fig. 1:**
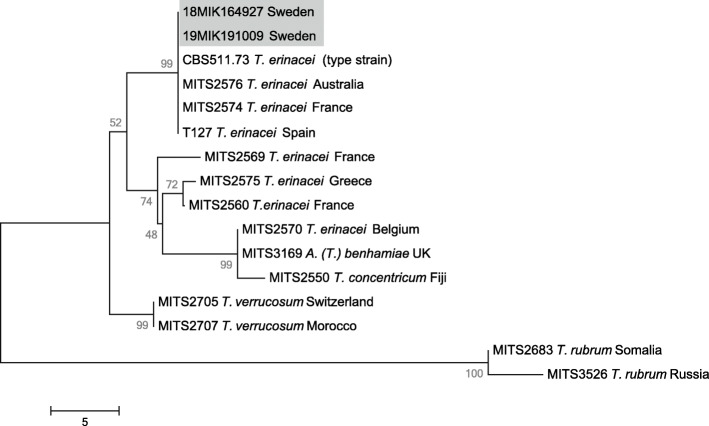
Neighbor-joining tree of ITS sequences from the two *Trichophyton* isolates recovered in the present study compared to ITS sequences from isolates of *T. erinacei* and closely related species. The scale is number of nucleotide differences in the 586 bp compared. Support from 10k bootstrap replicates is shown as a percentage at branch divisions. The more distantly related *T. rubrum* is included as an outgroup. The tree was created in MEGA 6

Twenty-three hedgehogs from Gotland County (n = 14), Skåne County (n = 6), and Uppsala County (n = 3) were investigated in the pilot-study and two, both originating from Gotland County, were found to carry dermatophytes. None of the sampled hedgehogs showed any visible signs of ongoing mycotic infection. The identified dermatophytes had identical Internal Transcribed Spacer (ITS) sequences and were identical to the *T. erinacei* type strain CBS 511.73 ITS sequence (GenBank AB105793) originally isolated from a European hedgehog in New Zealand (Fig. 1). The two ITS sequences were also identical to those of several *T. erinacei* isolates recovered from pet hedgehogs in Spain [24], represented in Fig. 1 by the isolate T-127 (GenBank KU852597), as well as additional *T. erinacei* isolates from other countries.

**Fig. 2:**
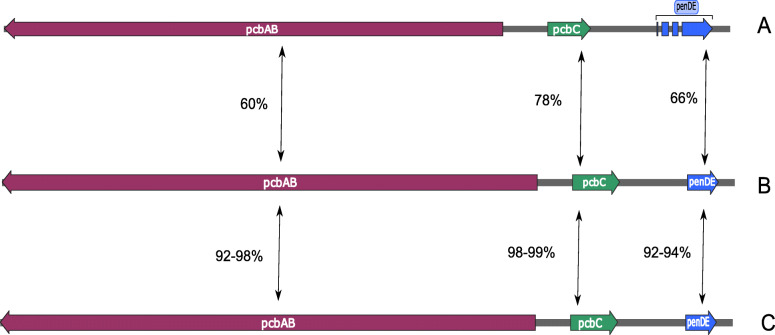
Comparison of the genes *pcb*AB, *pcb*C and *pen*DE in penicillin biosynthesis gene cluster (PGC). *T. erinacei* isolates from Swedish European hedgehogs (**B**) against the PGC in *Penicillium chrysogenum* Wisconsin 54-1255 strain (**A**) and the PCG identified in *T. rubrum* (NZ_ACPH00000000.1), *T. violaceum* (LHPN00000000.1), *T. verrucosum* (NZ_ACYE00000000.1), *T. mentagrophytes* (BFBS00000000.1), *T. interdigitale* (AOKY00000000.1), *T. equinum* (ABWI00000000.1), *T. benhamiae* (ABSU00000000.1), *T. tonsurans* (ACPI00000000.1) (**C**). The percentages given represent the similarity on amino acid level compared to the *T. erinacei* hedgehog isolates

Both identified *T. erinacei* isolates were confirmed to carry the genes, *pcbAB*, *pcbC* and *pen*DE, which encodes penicillin biosynthesis enzymes (Fig. 2), and expression of the *pcbAB* and *pcbC* genes was confirmed by quantitative reverse transcription real-time PCR (RT-qPCR) and subsequent melt-curve analysis. The length and identity of the genes were identical between both isolates but differed from those in the *Penicillium chrysogenum* Wisconsin 54-1255 reference strain (Fig. 2). Gene sizes were as follows; 11280bp for *pcbAB*, 1002bp for *pcbC,* 651bp for *penDE* in *T. erinacei* isolates, while 11376bp for *pcbAB*, 996bp for *pcbC,* 1275bp for *penDE* in the Wisconsin 54-1255 (Fig. 2). Amino acid comparisons between the two isolates and Wisconsin 54-1255 showed 60%, 70%, and 66% similarity for *pcbAB*, *pcbC* and *pen*DE respectively (Fig. 2). Similar comparisons to *Trichophyton* species, listed in Table 1, showed similarities of 92-98%, 98-99% and 92-94% for *pcbAB*, *pcbC* and penDE respectively.

**Table 1:** Trichophyton species and their respective reference IDs used to compare penicillin biosynthesis genes

*Trichophyton species*	Refseq ID/ INSDC ID
*T. rubrum*	NZ_ACPH00000000.1^a^
*T. violaceum*	LHPN00000000.1
*T. verrucosum*	NZ_ACYE00000000.1^a^
*T. mentagrophytes*	BFBS00000000.1
*T. interdigitale*	AOKY00000000.1
*T. equinum*	ABWI00000000.1
*T. benhamiae*	ABSU00000000.1
*T. tonsurans*	ACPI00000000.1

^a^Refseq ID

### Identification of benzylpenicillin

**Fig. 3:**
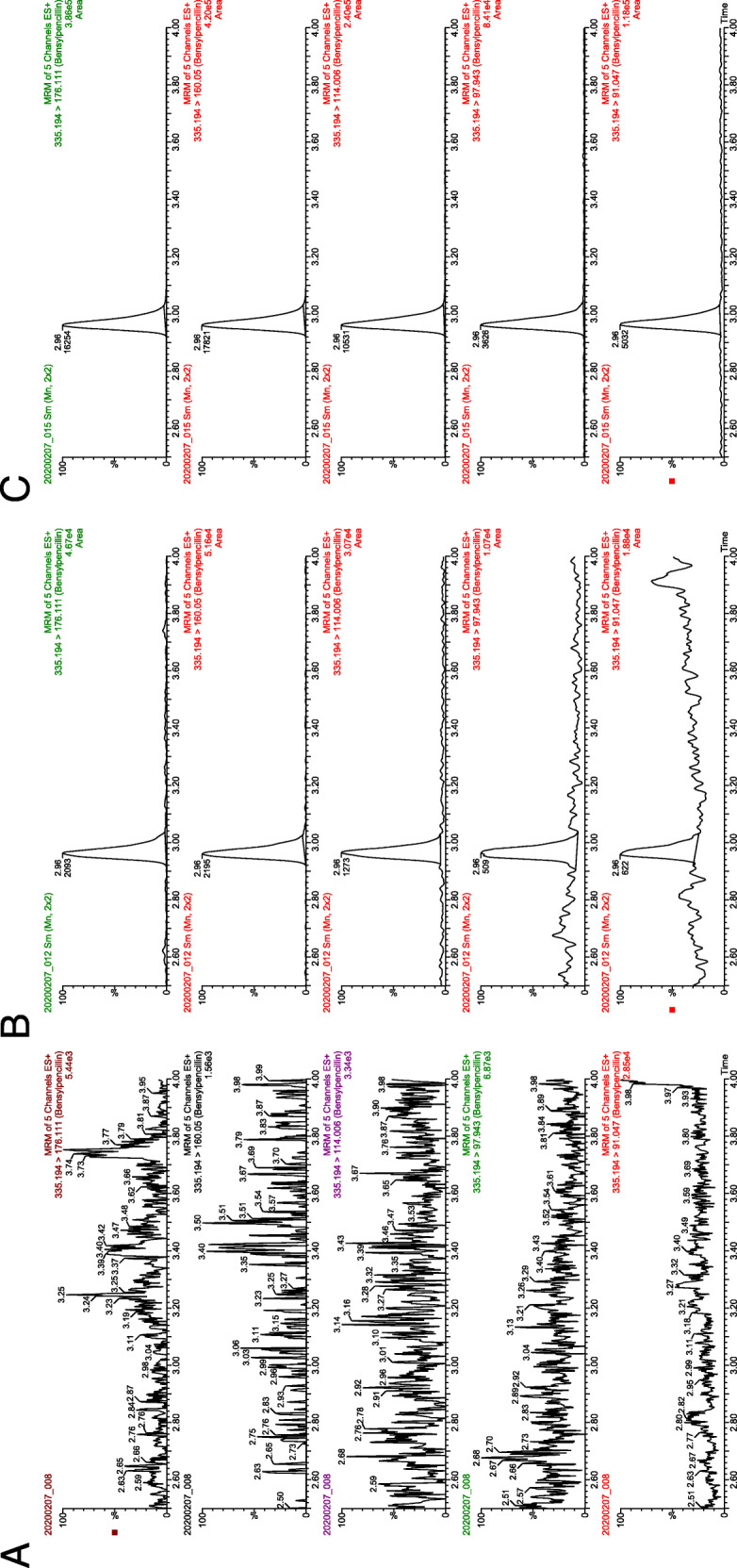
Mass chromatograms from UHPLC-MS/MS analyses. The characteristic SRM transitions m/z 335 [M+H]^+^ > 91, 335 [M+H]^+^ > 98, 335 [M+H]^+^ > 114, 335 [M+H]^+^ > 160 and 335 [M+H]^+^ > 176. **a** blank control, **b** sample 18MIK164927 and **c** reference standard of benzylpenicillin

In the mass chromatograms from ultra-high-performance liquid chromatography-tandem mass spectrometry (UHPLC-MS/MS) analyses (Fig. 3a–c), the characteristic daughter ion transitions m/z 335 [M+H]^+^ > 91, 335 [M+H]^+^ > 98, 335 [M+H]^+^ > 114, 335 [M+H]^+^ > 160 and 335 [M+H]^+^ > 176 are shown. The blank control (Fig. 3a) showed no chromatographic peaks but the sample 18MIK164927 (Fig. 3b) peaks were observed in all transitions at the retention time 2.96 min. These results agreed with those of the benzylpenicillin reference standard (Fig. 3c). In addition, the peak area ratios for the daughter ions were compared and these results were also consistent. The second isolate 19MIK191009 was also analyzed and the result from this analysis indicated presence of benzylpenicillin, but the peak responses were too weak for a definite identification.

### Recovery and characterization of *mec*C*-*MRSA

**Fig. 4:**
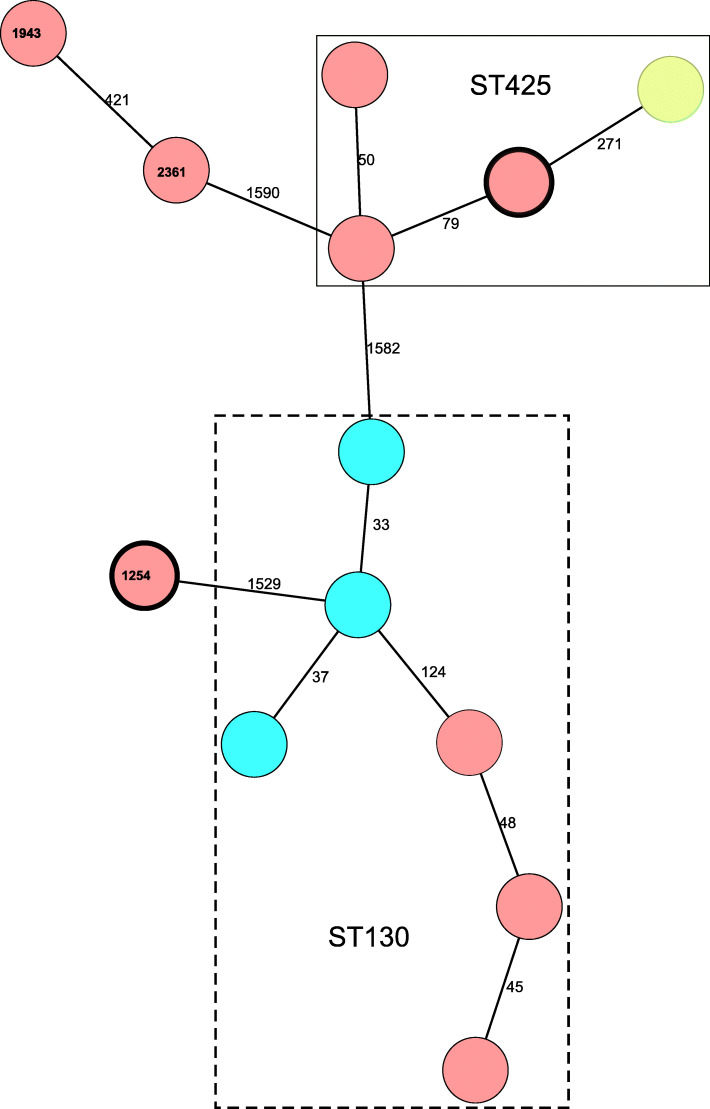
Minimum spanning tree (MST) of core genome MLST (cgMLST) relationship of methicillin resistant *S. aureus* carrying the *mec*C gene (*mec*C-MRSA) from European hedgehogs in Sweden. The cgMLST is based on target genes extracted from the *mec*C-MRSA genome LGA251, accession number FR821779, using the SeqSphere + software, which identified 1915 alleles present in all compared isolates. Nodes represent isolates (one isolate per node), Numbers in nodes denotes the multi-locus sequence type (7-MLST) as defined by the scheme at https://pubmlst.org/ and colours represent the origin of the isolate, Gotland County (Red) and Skåne County (Blue). Isolates encompassed in the solid box belong to ST425 while those in dashed box belong to ST130. Numbers on the lines between isolates indicate differences in alleles between isolates and line lengths are based on the allele differences in a logarithmic scale. Nodes marked in bold are *mec*C-MRSA isolates co-occurring with benzylpenicillin-producing *T. erinacei* isolates. The *mec*C-MRSA reference strain LGA251 (yellow) belonging to ST425, isolated from a dairy cow bulk milk sample in UK [6], is included in the figure for comparison

Of the 23 sampled hedgehogs 14 (60%) carried *mec*C-MRSA with 11 originating from Gotland county and 3 from Skåne County, with no MRSA positive hedgehogs found in Uppsala County. Because two isolates were lost at the laboratory during handling twelve isolates were available for genome sequencing. Both hedgehogs from Gotland positive for *T. erinacei* also carried *mec*C-MRSA which belonged to sequence types (ST) ST425 and ST1254. In total 5 different STs were identified, with the three isolates from Skåne County belonging to ST130 and the remaining isolates from Gotland County belonging to ST130 (n = 6), ST425 (n = 2), ST1943 (n = 1) and ST2631 (n = 1) (Fig. 4). Except for ST1943 and ST2361 that differed in one allele all recovered STs differed in ≥5 alleles. Using core-genome multi-locus sequence typing (cgMLST) and visualizing the difference in the number of alleles between isolates using a minimum spanning tree based on 1915 alleles, it was shown that the five STs differed significantly from each other (Fig. 3). Furthermore, there were >32 allele differences within each ST (Fig. 4). All *mec*C-MRSA isolates carried the staphylococcal cassette chromosome *mec* (*SCCmec*) type XI, *bla*Z_LGA251_, and were negative for PVL.

## Discussion

In the current study we could show that Swedish hedgehogs can carry benzylpenicillin producing *T. erinacei*. However, it was suggested already in the 1960s that *T. erinacei*, then called *T*. *mentagrophytes* var. erinacei, potentially could produce a penicillin-like substance [23]. This 1960s study was however limited in that it only showed that the investigated substance inhibited growth of penicillin-sensitive *S. aureus* strains and that the activity of the substance was hampered by penicillinase. In our study, genome sequencing was used to confirm the presence of the penicillin biosynthesis genes in the recovered *T. erinacei* isolates and RT-qPCR was used to show that the genes were expressed. In addition, UHPLC-MS/MS verified that the isolate 18MIK164927 produced benzylpenicillin (Fig. 3), but that the second isolate 19MIK191009 only had a weak peak response (data not shown). The weak peak response for 19MIK191009 was not unexpected as fungal strains grown under standard conditions may only produce small quantities of benzylpenicillin since production is normally triggered by stress or environmental conditions. As an example, commercial production of benzylpenicillin in *Penicillum* species relies on extensive genetic and environmental optimization with current strains producing 100.000 times more penicillin than the *P. notatum* strain originally described by Fleming [25]. Other studies have also confirmed production of antimicrobials by dermatophytes from other settings, but this is to our knowledge the first time for *T. erinacei* [26]. Albeit only two hedgehogs carried *T. erinacei* previous studies on hedgehogs have shown that this dermatophyte species is widespread among hedgehogs, especially during the winter months [27–29]. The higher incidence of *T. erinacei* during winter months, when hedgehogs hibernate, could explain the lower number of positive samples in the current study, since all hedgehogs included were sampled in spring to early autumn.

The identification of penicillin-producing *T. erinacei* in hedgehogs is important since this dermatophyte might influence the skin bacterial flora of the hedgehogs by imposing a selection pressure on or working in a symbiotic relationship with the flora. The selective pressure, or symbiosis, could facilitate the emergence of resistant variants of bacteria such as *mec*C-MRSA, and both hedgehogs that carried penicillin-producing *T. erinacei* also carried *mec*C-MRSA (Fig. 4). A previous study has however described that the PBP2c produced by *mec*C has lower affinity for penicillin than the PBP2a produced by *mec*A [28], which could contradict the hypothesis of a possible connection between *mec*C-MRSA and penicillin-producing *T. erinacei*. However, the selection of *mec*C-MRSA on hedgehogs could be influenced by the presence of the *bla*Z_LGA251_ gene which produces beta-lactamase and is in close proximity to the *mec*C on the SCC*mec* XI. In the current study, as in previous ones, the isolates were all positive for the SCCmec XI and carried *bla*Z_LGA251_. In opposition to *mec*A-MRSA it has also been shown that broad-spectrum beta-lactam resistance for *mec*C-MRSA is due to the combined presences of *mec*C and *bla*Z_LGA251_ on the SCC*mec* XI [30]. It is also worth to note that *mec*A-MRSA might also have evolved due to selection pressure from penicillin rather than through the more modern methicillin [31].

In accordance with previous studies, we found that *mec*C-MRSA is common, 60%, among wild hedgehogs [8, 11]. We were also able to show that the *mec*C-MRSA isolates belonged to five distinct 7-MLST types and that it was a high diversity between and within STs based on cgMLST (Fig. 4). The high diversity indicates that occurrence in hedgehogs is not due to nosocomial spread at rescue centers or a local endemic spread fueled by release of animals. In addition, the recent Danish study showed the same high *mec*C-MRSA incidence on hedgehogs not connected to rescue centers [11]. Previous studies also emphasized the high *mec*C-MRSA occurrence and diversity in this animal species, but based on *spa*-types rather than high-resolution genome sequencing [8, 11]. Furthermore, the STs identified in this study have all previously been shown to be connected to *mec*C-MRSA except for ST1254, and all isolates in this study carried the SCC*mec* XI, which is the SCC*mec* linked to *mec*C [16, 17]. The *mec*C-MRSA associated STs identified in this study, and others, also overlaps with those identified from clinical cases in humans, dairy cattle, goats, and companion animals in Sweden [18, 32]. These combined studies therefore all lend further support to the suggestion that *mec*C-MRSA could be a wildlife associated MRSA (WA-MRSA), rather than a LA-MRSA, with the main reservoir being European hedgehogs [17]. If European hedgehogs, which primarily are native to Northern and Western Europe, are the main reservoirs for *mec*C-MRSA it would also help explain why *mec*C-MRSA mainly has been found in Europe [16, 17]. However, it is worth noticing that other wildlife reservoirs might exist as a recent Spanish study showed a relatively high incidence of *mec*C-MRSA in wild rabbits [33].

To understand the occurrence and evolution of *mec*C-MRSA and its connection to penicillin-producing *T. erinacei* and to hedgehogs, additional studies are warranted as the current pilot-study is limited in the number of samples and isolates investigated. Such studies should be; i) more extensive sampling of hedgehog populations from wider geographic areas and during hibernation, as well as pet hedgehogs, to ascertain a statistically viable correlation between *mec*C-MRSA and penicillin producing *T. erinacei*, ii) investigation of the influence of the penicillin-production of *T. erinacei* on *mec*C-MRSA, *mec*A-MRSA, and penicillin-sensitive *S. aureus* strains in-vitro, iii) investigation of other wildlife species for occurrence of *mec*C-MRSA to establish if occurrence is exclusively linked to hedgehogs and iv) determination of resistance profiles of additional microflora from hedgehogs that are carriers of penicillin-producing *T. erinacei* which would provide an insight on the mechanics of antimicrobial resistance in nature.

### Conclusions

The current study shows that European hedgehogs in Sweden can carry benzylpenicillin-producing *T. erinacei* and that the hedgehogs that carry these dermatophytes also carries *mec*C-MRSA. In addition, the study confirms the high occurrence and diversity of *mec*C-MRSA in the European hedgehog population. The results emphasize the further need to study the association, host-adaption and potential shared evolution of *mec*C-MRSA, *T. erinacei* and European hedgehogs.

## Methods

### Collection and sampling of hedgehogs

In July 2018, custodians at two rescue centres in Gotland County and Skåne County were invited to send dead hedgehogs to the National Veterinary Institute (SVA) for the study. Included hedgehogs were euthanized due to injury or illness or had already died of natural causes upon arriving at the rescue centres. Euthanasia was performed by trained personal through intraperitoneal injection with pentobarbital according to Swedish legislation. In addition to the two rescue centres the study participants also collected dead hedgehogs found as roadkill in Uppsala County. Ethical approval according to national legislation (SJVFS 2015:38) was not required since samples were collected from wildlife where the cause of death, natural or euthanized, was unrelated and occurred prior to inclusion in the study. The study adhered to the Animal Research: Reporting of In Vivo Experiments (ARRIVE) guidelines.

Sampling was carried out between July 2018 and ended in October 2019. Sampling during winter months was not feasible since the animals were in hibernation. All hedgehogs were shipped to the laboratory within 24–48h of death and subsequently sampled within 24 h. For dermatophytes, quills and skin scrapings were taken and for MRSA, oral cavity, nostril, skin, and perineum of hedgehogs were swabbed using Copan Eswabs^TM^ (Copan).

### Fungi cultivation and identification

Samples were inoculated on Sabouraud agar (SAB) with Chloramphenicol, pH 7, (National veterinary Institute, SVA) and modified Dixon agar (SVA), and then incubated at 27 °C for 15 days. Cultures were macroscopically analysed for dermatophytes and all fungi cultures characteristic of dermatophytes were further subjected to microscopy. Prior to microscopy, a drop of MYCETBLUE^TM^ stain was placed at the centre of a microscope glass slide and a piece of cellophane tape was used to collect and apply a culture in the stain on the glass slide. The slide was then incubated at room temperature for 15 min and viewed under a light microscope at 100-400X magnification to verify presence of dermatophytes based on the morphology of macro- and microconidia.

### MRSA isolation and identification

Copan Eswabs^TM^ (Copan) were briefly vortexed, and 0.2 mL of the suspension was added to 4.8 mL trypticase soy broth (TSB) supplemented with 4% NaCl, 1% mannitol, and 10 mg/L aztreonam, and incubated overnight at 37 °C. To select for MRSA, 10 μL of the overnight culture was streaked on MRSA 2 Brilliance agar (Oxoid), Mannitol salt agar (SVA) and horse blood agar (SVA), respectively, and plates were incubated at 37 °C overnight.

Suspected MRSA-colonies were subjected to Matrix Assisted Laser Desorption and Ionization- Time of flight (Bruker Maldi-TOF Biotyper System) to confirm species*,* and presences of *mecA/mecC/*PVL*/nuc* was confirmed using a real-time quadruplex PCR [34]**.**

### DNA extraction, sequencing, and bioinformatics

Verified dermatophytes were cultured on SAB without antibiotics at 30 °C for 14 days to obtain pure cultures. Colonies of the pure cultures were homogenised by a mortar and pestle in liquid nitrogen followed by total DNA extraction using DNeasy Plant Mini Kit (Qiagen) according to manufacturer’s instructions.

MRSA isolates were cultured on horse blood agar and genomic DNA was extracted from a 1 μL loopful of colonies using EZ1 tissue kit (Qiagen) on a EZI advanced XL (Qiagen).

DNA from dermatophyte and MRSA were prepared using Nextera XT DNA library preparation kit (Illumina) and genome sequencing was performed on a MiSeq Illumina platform, as 2x250 bp paired-end reads aiming for a minimum average coverage of 40X. The quality of sequence data was analysed using the FastQC program [35]**.**

Sequence data from *S. aureus* were subsequently run through an assembly, species determination, and antibiotic resistance classification pipeline composed of Trimmomatic 0.36 [36], SPAdes 3.5.0 [37], Pilon 1.2.3 [38], Kraken using mini-Kraken 8GB-database [39], and ARIBA [40] using the ResFinder database (http://www.genomicepidemiology.org/). *SCCmec* cassette typing was performed using SCCmecFinder (https://cge.cbs.dtu.dk). 7-MLST and core genome MLST (cgMLST) was performed using the Ridom SeqSphere+ software [41], with the genome of LGA251, accession number FR821779 used to define an ad-hoc cgMLST scheme with default settings in SeqSphere+.

For the assembly of dermatophyte sequencing data, the same pipeline as for MRSA was used but excluding ARIBA and Kraken. To identify the dermatophyte species, the ITS region of *Trichophyton rubrum* (refseq: NZ_ACPH00000000.1) was used in a BLAST+ search against the dermatophyte assemblies. Matches to ITS with the highest score, coverage, identity, and lowest E-value were extracted from respective contigs and used in species identification performed by matching against the ISHAM Barcoding Database (http://its.mycologylab.org/). Reference *T. erinacei* ITS sequences from the ISHAM Barcoding Database were retrieved, sequences were aligned by the ClustalW algorithm and their relatedness investigated by the Neighbor joining algorithm in Mega 6 [42] excluding positions with gaps and otherwise with default settings. The resulting tree was evaluated by generating 10,000 bootstrap replicates.

### Identification and expression of penicillin biosynthesis genes (PGs)

To identify the penicillin biosynthesis gene cluster (PGC) containing the genes, *pcbAB*, *pcbC* and *penDE* in the recovered *T. erinacei*, the PGC sequence from the *Penicillium chrysogenum* isolate Wisconsin54-1255 (accession number EF601124.1) was used as reference for a BLAST+ analysis of the generated contigs. The full ORFs for the corresponding homologues in the analysed assembly were extracted from a contig producing BLAST hits for all three genes using NCBI ORFfinder tool (https://www.ncbi.nlm.nih.gov/orffinder/) and Snapgene (GSL Biotech, snapgene.com). Gene orthologues from available *Trichophyton* species (Table 1) were compared to genes from the recovered strains at amino acid level using the NCBI BlastP suite.

To evaluate expression of the penicillin biosynthesis genes, colonies from a 14-day pure culture were homogenised by a mortar and pestle in liquid nitrogen followed by extraction of total RNA using Trizol (Thermofisher) and phenol-chloroform extraction method. The resultant RNA was treated with DNase 1 (Thermo Scientific™) according to manufacturer’s instructions. Expression of two genes, *pcbAB* and *pcbC* was investigated using iTaq Universal One-Step RT-qPCR Kit, in triplicates with in-house deigned primers sets using Primer3 with default settings (http://bioinfo.ut.ee/primer3-0.4.0/) (Table 2)*.* These primers were designed only for use on pure cultures and were not evaluated for specificity in other contexts. Dnase treated RNA, fungi genomic DNA (used as positive control), were analysed and RNA without the DNase treatment was used as a quality control for the efficiency of eliminating DNA remnants post extraction. The thermal cycling program was as follows; 10 min at 50 °C, 1 min at 95 °C followed by 40 cycles of 15 sec at 95 °C and 1 min at 60 °C subsequent melt-curve analysis of 60 °C–95 °C with 0.5 °C increment for 4 sec per step as an indicator to confirm the specific amplification of a single target consistent with the product produced in the positive control .

**Table 2:** Oligonucleotide primer sets for RT-qPCR

Gene	Primer label	Sequence	Amplicon size(bp)
*pcbAB*	pcbAB_F	5’-TCTGAGCGGGGAATCACTTT -3’	223
	pcbAB_R	5’-CAGACATTGCATCCAGGACG-3’	
*pcbC*	pcbC_F	5’-CGCCCATTCATCGTGTCAAA-3’	164
	pcbC_R	5’-ATCCTGCAAGTACTGGCCAT-3’	

### Identification of benzylpenicillin

Scrapings from 14-day cultures of each *Trichophyton* isolate were stored in absolute methanol at -80 °C awaiting chemical analysis. A blank control sample was included which was treated identical to the hedgehog samples. The methanol extract (supernatant) of each sample (40 μL) was diluted with 160 μL MilliQ-water, vortexed for 1 min and transferred to a vial. For substance identification and comparison, a prepared standard (1 ng/mL in methanol:MilliQ-water (20/80, v/v)) of a European pharmacopoeia reference material of benzylpenicillin sodium (certified reference standard, from Sigma-Aldrich Corporation, Germany) was used.

Benzylpenicillin analysis was performed with a UHPLC-MS/MS system composed of an Acquity UPLC coupled to a Xevo TQ-Sμ mass spectrometer (Waters Corporation, MA, USA) with an electrospray interface. The chromatographic system used consisted of a C18 guard column (Waters Corp.) and an Acquity BEH C18 column (2.1 x 100 mm, 1.7 μm) (Waters Corp.) both at 65 °C. The mobile phase consisted of A) 0.1 % formic acid in water and B) methanol and it was delivered as a gradient. The gradient started at 30% B to 0.40 min, then continued with a linear increase over time to 80% B at 4.00 min, and 95% B at 4.50 min. At 5.50 min the gradient switched to 10% B and was held constant for 1.5 min for equilibration. The flow rate was 0.400 mL/min and the injection volume 10 μL. The analyte was analysed with a positive capillary voltage of 1.2 kV and a cone voltage of 18 V. The desolvation temperature was 500 °C and desolvation gas flow was 1000 L/h. The ionization was positive electrospray and benzylpenicillin was detected as [M+H]^+^. The mass spectrometric analysis mode was selected reaction monitoring (SRM) using daughter ion transitions characteristic for benzylpenicillin. The SRM transitions for the qualitative detection were m/z 335 > 91, m/z 335 > 98, m/z 335 > 114, m/z 335 > 160 and m/z 335 > 176 (collision energy 44 eV, 32 eV, 30 eV, 10 eV and 12 eV respectively). The results were evaluated using the software TargetLynx (Waters Corp.). The evaluation of data was done using the EC. 657/2002. Commission Decision 2002/657/EC as guideline.

## Data Availability

The data and isolates generated during the current study are available from the authors upon reasonable request. Sequence reads *T. erinacei* and *mec*C-MRSA isolates have been deposited in the European Nucleotide Archive under the accession number PRJEB37151 and PRJEB37163, respectively.

## Abbreviations

cgMLST: core-genome Multi-locus sequence typing
ITS: Internal Transcribed Spacer
MRSA: Methicillin-resistant Staphylococcus aureus
PCR: Polymerase Chain Reaction
PGC: penicillin biosynthesis gene cluster
RT-qPCR: Reverse transcription quantitative PCR
SAB: Sabouraud agar
SCCmec: Staphylococcal chromosomal cassettes mec
SRM: Selected reaction monitoring
ST: Sequence types
UHPLC-MS/MS: Ultra-high-performance liquid chromatography-tandem mass spectrometry
MLST: Multi-locus sequence typing
